# The Relative Influence of Family and Neighborhood Factors on Child Maltreatment at Critical Stages of Child Development

**DOI:** 10.3390/children9020163

**Published:** 2022-01-27

**Authors:** Kathryn Maguire-Jack, Susan Yoon, Yujeong Chang, Sunghyun Hong

**Affiliations:** 1 School of Social Work, University of Michigan, Ann Arbor, MI 48109, USA; yujeongc@umich.edu (Y.C.); hshong@umich.edu (S.H.); 2 College of Social Work, The Ohio State University, Columbus, OH 43210, USA; Yoon.538@osu.edu

**Keywords:** child maltreatment, path analysis, neighborhoods, families, risk and protective factors

## Abstract

This study examines the impact of family and neighborhood factors on physical and psychological abuse across three developmental stages of children: early childhood (age 3), young school age (age 5), and middle childhood (age 9). Data from the Fragile Families and Child Wellbeing Study, a longitudinal national cohort study of children from 20 urban U.S. cities, are used. Path analysis is employed to investigate the longitudinal relationships between family and neighborhood context variables and abuse risk, as well as the importance of different factors at key developmental stages. Economic hardship, maternal substance use, intimate partner violence, and exposure to community violence are found to be related to child abuse risk regardless of developmental stage, while maternal depression and neighborhood informal social control are found to have impacts only within certain child development stages. Findings suggest the need for early intervention and prevention strategies that specifically target economic hardship, poverty, intimate partner violence, and exposure to community violence.

## 1. Introduction

Experiences in childhood are critical to their long-term outcomes over their life course. Over the past several decades, researchers have discovered many critical aspects of the context in which a child grows that relate to their likelihood of experiencing child maltreatment [1]. These include factors at various levels of the social ecology, with the characteristics of the child, parents, families, and neighborhoods affecting the likelihood that maltreatment occurs [2]. Although researchers have examined the influence of such factors occurring at these various ecological levels, there is limited understanding of the relative influence of these different factors at different developmental stages of children and the extent to which the effect of these factors in early periods of development persist into later ages. The current study addresses this gap in the literature by specifically modeling factors about the family, school, and neighborhood at three critical periods of child development—early childhood (age 3), young school age (age 5), and middle childhood (age 9).

### 1.1. Theory

The causes of child maltreatment are complex. There are a multitude of theories that seek to understand why parents maltreat their children. This work is guided by ecological systems theory [3], which suggests that individuals are influenced by factors at multiple levels and that these factors coalesce to affect health and development in people. This study is also guided by the developmental psychopathology perspective [4,5]. Similar to ecological systems theory, this perspective underscores the significance of understanding developmental outcomes through an interplay between an individual and one’s surrounding contexts (e.g., family, school, neighborhood). Developmental psychopathology further stresses that both the nature (e.g., multi-level contexts) and timing (e.g., developmental timing) of an experience are critical in determining one’s developmental pathways and outcomes. The theory emphasizes that individuals’ development and health can be understood properly only by examining developmental history, changing contexts, and evolving outcomes over time, highlighting the importance of applying a developmental lens in health science research.

### 1.2. Family Context

The family context is one of the most proximal and influential environmental factors related to child maltreatment. At the family level, key factors such as family socioeconomic (SES) status and parental characteristics (e.g., parental behavioral health) can significantly impact the likelihood of child maltreatment. Poverty and child maltreatment are closely linked [6]. Parents who are living with limited economic resources may have decreased ability to meet their children’s basic needs. Additionally, economic hardships are associated with increased stress in parents, which is related to an increased risk for harsh parenting [7,8,9,10].

Intimate partner violence (IPV) often co-occurs with child maltreatment [11], with children living in homes with IPV being 2.5 times more likely to experience physical abuse and 9.5 times more likely to experience psychological abuse [12]. IPV can create dangerous situations for children, who may try to intervene to protect the survivor. IPV also causes a great deal of stress for survivors, which may, in turn, influence their parenting behaviors, both in terms of harsher parenting practices as well as lower levels of supervision and care. Finally, in homes where IPV is present, the intimate partner abuser may also be committing violence against the children.

Depression can increase fatigue and make it difficult for individuals to conduct their normal daily activities. Among parents, depression can inhibit a parent’s ability to properly care for their children. It may also contribute to increased levels of harsh parenting because parents may be so focused on their own mental health issues that they are less sensitive to children’s needs and therefore may be more reactive and use harsher discipline than they typically would. Parental depression and emotion dysregulation is associated with a greater likelihood of using an authoritarian parenting style [13]. Prior research has found depression to be linked to an increased risk for psychological abuse [14], physical abuse [1], and neglect [1].

Finally, substance use may impact the ways in which parents care for their children. Parents who struggle with substance-use disorder may be emotionally and physically unavailable to meet their children’s basic needs. Additionally, children’s basic needs may go unmet because of financial resources being redirected to the substance-use problem. Substance use is associated with lower inhibitions [15], and parents, therefore, may react more harshly to their children than they would under normal circumstances. Parents who use substances are at greater risk for maltreating their children [16].

### 1.3. Neighborhood Context

While the direct family context impacts the likelihood that parents will maltreat, parents also interact with their direct neighborhoods and communities that may also play an important role. There are critical process factors within neighborhoods that relate to the relationships and interactions that residents have with each other. Social cohesion relates to the bonds and ties between neighbors and captures the social relationships they have with each other [17]. Informal social control relates to the willingness of neighbors to intervene in problematic social situations on behalf of the common good [17]. Parents who have trusting relationships with their neighbors and feel as though their neighbors are looking out for the best interests of their children may feel supported, and therefore less stressed. They also might be able to rely on their neighbors for favors such as emergency childcare or food assistance, which would impact their ability to meet their child’s basic needs. Social cohesion and social control have been found to be protective against child maltreatment [18].

Families are also impacted by the violence occurring within their communities. Children who live in communities with high levels of violence may require higher levels of supervision compared to children in communities in which it is safe for them to play outdoors. Witnessing violence within the community may also contribute to higher levels of stress in parents, which may then translate into harsher parenting practices. Prior research has demonstrated a link between community violence and abusive parenting [19].

### 1.4. Developmental Considerations

This study is focused specifically on the relative importance of the family and neighborhood contexts in child maltreatment across key developmental periods in children—early childhood (age 3), young school age (age 5), and middle childhood (age 9). We focus on these developmental periods because children’s ecological systems become larger over time. In early childhood, children are neither in school nor old enough to be left alone, and they are very reliant on their caregivers. Because parents may experience more difficulty traveling outside their neighborhoods to access goods and services, they may be more reliant on the networks within close proximity to their home. Once children enter school, parents may become more engaged outside of their direct neighborhoods due to their children’s school and extracurricular activities. As such, the quality of their direct proximal neighborhood may have less of an impact on their parenting behaviors as their personal networks grow and have a larger influence. However, it is anticipated that neighborhoods still play a crucial role at this developmental stage, in which children tend to interact more often with other children within the neighborhood, increasing the contacts between parents and other residents, and children still require supervision and childcare. In middle childhood, the impact of neighborhood on child maltreatment is expected to continue to lessen as children grow and make more school friends and expand their own networks; parents may have less frequent interactions with their neighbors.

### 1.5. Current Study

The current study extends prior research examining factors of the family and neighborhood context related to child abuse by exploring the relative importance of these two contexts of children at key developmental stages. We specifically ask the following research questions: (1) What is the relative influence of the family and neighborhood context on child abuse at ages 3, 5, and 9? (2) What is the longitudinal relationship between the factors and child abuse across these three ages?

## 2. Methods

### 2.1. Data and Sample

The data for the current study is from the Fragile Families and Child Wellbeing Study (FFCWS). FFCWS is a longitudinal birth-cohort study of 4898 children from 20 urban U.S. cities and was deemed human subjects research by the University of Michigan Health Sciences and Behavioral Sciences Institutional Review Board (FWA 00004969) and approved on 7 October 2019. Parents were interviewed at the time of the child’s birth and then again when the focal child was age 1, 3, 5, 9, and 15. The current study focused on the waves of data collection when children were ages 3, 5, and 9.

### 2.2. Measures

#### 2.2.1. Key Dependent Variables

Child maltreatment was assessed using two subscales of the Conflict Tactics Scale—Parent–Child Version (CTS-PC; [20]): psychological aggression and physical assault. In the FFCWS, a shortened version of this scale was used, with questions representing severe physical abuse being removed (e.g., burning on purpose). The scales used included five questions each and were asked how many times in the past year a behavior happened. Responses were coded as “once”, “twice”, “3–5 times”, “6–10 times”, “11–20 times”, “more than 20 times”, “yes but not in the past year”, and “this has never happened”. An example item from the psychological aggression subscale is: “Called him/her dumb or lazy or some other name like that.” An example item from the physical assault subscale is: “Hit him/her on the bottom with something like a belt, hairbrush, a stick, or some other hard object.” As recommended by the scale developers [20], we coded responses of “once” as 1, “twice” as 2, “3–5 times” as 4, “6–10 times” as 8, “11–20 times” as 15, “more than 20 times” as 25, and both “yes but not in the past year” and “this has never happened” as 0. We then summed across the two subscales at each of the three waves to get a total count of maltreatment events at that time point. Across waves, the interitem reliability for psychological aggression ranged from 0.52 to 0.62 and 0.61 to 0.70 for physical assault. Although these numbers are below the commonly accepted levels for Cronbach’s alpha, it is common for maltreatment measures to have low interitem reliability due to caregivers choosing some abusive behaviors and not others.

#### 2.2.2. Key Independent Variables—Family Context

To understand the importance of the family context on child maltreatment, we examined the characteristics of the parents and the relationships within the family. Economic hardship was measured using a battery of questions in which participants were asked whether they had experienced a variety of material hardships related to finances in the past year. At age 3, FFCWS included 10 questions of this nature, at age 5 there were 13, and at age 9 there were 10. An example question across all three waves is: “In the past 12 months, did you receive free food or meals because there wasn’t enough money?”. The three additional questions included at age 5 were: “Were the children ever hungry because you just couldn’t afford more food?”; “Have you cut back on buying clothes for yourself because there wasn’t enough money?”; and “Have you worked overtime or taken a second job because there wasn’t enough money?”. At each wave, we took the sum of the number of “Yes” responses for a total count of economic hardships. The interitem reliability for this scale ranged from 0.65 to 0.72 across all three waves.

Intimate partner violence was measured using a subset of questions from the revised Conflict Tactics Scale 2 [21]. Seven questions were asked of mothers about the biological father and their current partner. An example item is: “He hits you with a fist or an object that could hurt you.” Response options included “never”, “sometimes”, or “often”. We coded “never” as 0, “sometimes” as 1, and “often” as 2, and took the mean for the whole scale at each wave. The interitem reliability for this scale ranged from 0.60 to 0.67.

Maternal depression was assessed by the Composite International Diagnostic Interview [22]. Mothers who met diagnostic criteria for depression were assigned a 1, while those who did not were assigned a 0. This was measured at all three waves.

Maternal substance use was assessed at all three waves through questions regarding alcohol and illicit drug use. Regarding illicit drug use, if the mother reported any use of illicit drugs in the past 30 days, drug use was assigned a 1. For alcohol use, if the mother reported that drinking or being hungover had interfered with their work at school, a job, or home at least once in the past year, she was assigned a 1. Mothers that did not have illicit drug use or problematic alcohol use were assigned a 0.

#### 2.2.3. Key Independent Variables—Neighborhood Context

To understand the neighborhood environment of the child, we examined collective efficacy, neighborhood poverty rate, and exposure to violence. Collective efficacy was measured using two subscales—social cohesion and informal social control [17]. Social cohesion is intended to measure the trust and bonds between neighbors and was measured on a Likert scale ranging from “Strongly disagree” to “Strongly agree”. An example item from this subscale is: “People around here are willing to help their neighbors”. At age 3, there were five items included, but beginning at age 5, FFCWS cut one question: “People in this neighborhood can be trusted”. We reverse coded two items that were scaled in the opposite direction and scaled all variables such that a higher number represented a higher level of agreement with the items or more social cohesion. The interitem reliability for this scale ranged from 0.76 to 0.81 across the three waves. Informal social control measures the willingness of neighbors to intervene in a variety of social problems occurring within the neighborhood. This subscale included five items at all three waves and was measured on a Likert scale ranging from “Very unlikely” to “Very likely”. We scaled all variables such that a higher number represented a greater likelihood reported or a higher level of informal social control. An example item from this subscale is: “How likely would your neighbors be to intervene if children were skipping school and hanging out on a street corner?”. The interitem reliability for this measure ranged from 0.87 to 0.88 across the three waves. For both of these measures, we took the mean of the entire subscale at each time point.

Exposure to violence was measured with a series of seven questions. Participants were asked how many times they had witnessed a variety of violent acts (e.g., “In the past year, about how many times did you see someone else get shot at by someone?”). The scale provided included the following options: “never”, “once”, “2–3 times”, “4–10 times”, and “more than 10 times”. Participants were instructed to report on violence carried out by someone outside of their direct circle of family and friends and to not consider violence seen on television or movies. The items were assigned a 0 if they reported “never”, a 1 if they reported “once”, a 2 if they reported “2–3 times”, a 3 if they reported “4–10 times”, and a 4 if they reported “more than 10 times”. At age 9, FFCWS dropped four of the seven exposure to violence questions: “In the past year, about how many times were you hit, slapped, punched, or beaten up by someone?”; “In the past year, about how many times were you attacked with a weapon by someone?”; “In the past year, about how many times were you shot at by someone?”; and “In the past year, about how many times did you see someone get killed because of violence by someone?”. We took the mean of the available items at each wave. The interitem reliability for this scale ranged from 0.70 to 0.74 across the three waves.

#### 2.2.4. Control Variables

We controlled for three variables that have been shown in prior research to be related to our independent and dependent variables: maternal age, marital status, maternal education, child sex, and child race. Maternal age was measured continuously in years. Marital status was assessed at each wave as a dichotomous indicator, representing whether the biological mother and father were married. Maternal education was included at each wave as a dichotomous variable indicating that the mother had a high school degree (or equivalent) or higher education. Child sex was measured dichotomously, with a 1 indicating that the child was male and 0 indicating that the child was female. Child race was measured with a series of dichotomous variables for White, Black, Hispanic, some other race, or multi-racial.

### 2.3. Data Analysis

Before performing the primary analyses, we performed a preliminary analysis to examine variable distributions, invalid data values, and influential outliers. Next, we conducted path analysis with time-varying covariates to examine the concurrent and lagged effects of the family and neighborhood context on child maltreatment at ages 3, 5, and 9. We estimated path analysis models where child maltreatment at ages 3, 5, and 9 were outcomes, family and neighborhood context factors at ages 3, 5, and 9 were focal predictors, and demographics at age 3 were control variables. We regressed outcome variables on time-varying predictors by specifying both concurrent paths and lagged paths. By estimating lagged paths, we were able to examine the extent to which the effects of the early context on child maltreatment outcomes persisted over time. The outcome variables were also regressed on time-invariant control variables (i.e., maternal age, father and mother married, child sex, and child race). The model fit was evaluated using fit indices, with the Comparative Fit Index (CFI) and Tucker–Lewis Index (TLI) ≥ 0.95, root mean square error of approximation (RMSEA) ≤ 0.06, and the Standardized Root Mean Square Residual (SRMR) ≤ 0.05, indicating a good fit [23,24]. Data preparation and descriptive analyses were completed using STATA v.15, and the path analysis was conducted using Mplus v.8.6. Full information maximum likelihood (FIML) was used to handle missing data. FIML is considered less biased and more efficient than other methods (e.g., pairwise deletion, listwise deletion) to address missing data [25].

## 3. Results

### 3.1. Sample Characteristics

Table 1 summarizes the sample characteristics. A little over half of the children in the study were boys (52.2%). In terms of child race/ethnicity, 44.1% was Black/non-Hispanic, 16.0% White/non-Hispanic, 22.9% Hispanic, 2.0% multiracial, and 15.0% other race (American Indian, Asian, Native Hawaiian/ Pacific Islander). Mothers’ ages ranged from 15 to 43 years (mean age = 25.28, SD = 6.04). About 20.1% of the mothers had less than high school education, and 32.1% were married to the child’s biological father.

### 3.2. Family and Neighborhood Predictors of Physical Abuse and Psychological Abuse

**Figure 1:**
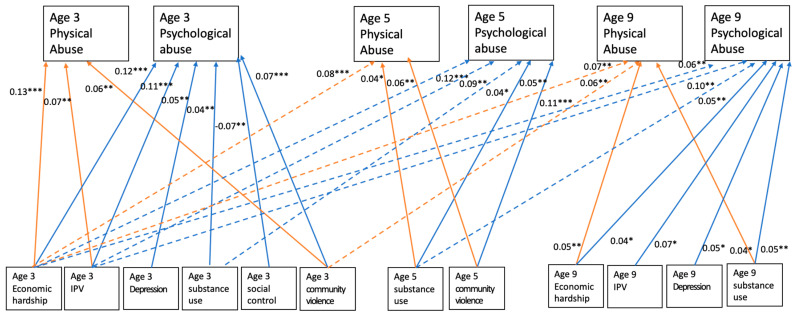
Predictors of physical abuse and psychological abuse at ages 3, 5, and 9. Note: Solid lines indicate concurrent associations and dashed lines indicate lagged effects. * *p* < 0.05, ** *p* < 0.01, *** *p* < 0.001. IPV = intimate partner violence.

The path model showed a good fit to the data: CFI = 0.995, TLI = 0.979, RMSEA = 0.013, 90% CI (0.008, 0.018), and SRMR = 0.008. At age 3, economic hardship (β = 0.13, *p* < 0.001), intimate partner violence (β = 0.07 *p* = 0.008), and exposure to community violence (β = 0.06, *p* = 0.001) were positively associated with physical abuse. At age 5, substance use (β = 0.04, *p* = 0.031) and exposure to community violence (β = 0.06, *p* = 0.001) were positively associated with physical abuse. At age 9, economic hardship (β = 0.05, *p* = 0.006) and substance use (β = 0.04, *p* = 0.031) were positively associated with physical abuse. In addition to the concurrent relationships at each age, several lagged effects were found. Economic hardship at age 3 was positively associated with physical abuse at age 5 (β = 0.08, *p* < 0.001) and age 9 (β = 0.07, *p* = 0.002). Additionally, exposure to community violence at age 3 was positively associated with physical abuse at age 9 (β = 0.06, *p* = 0.007). Family and neighborhood factors at age 5 did not have any lagged effects on physical abuse at age 9. Table 2 and Figure 1 summarize the concurrent and lagged effects of family and neighborhood contexts on child physical abuse.

In terms of child psychological abuse (see Table 3 and Figure 1), economic hardship (β = 0.12, *p* < 0.001), intimate partner violence (β = 0.11, *p* < 0.001), depression (β = 0.05, *p* = 0.002), substance use (β = 0.04, *p* = 0.009), and exposure to community violence (β = 0.07, *p* < 0.001) at age 3 were all significantly and positively associated with psychological abuse at age 3. Additionally, age three social control was negatively associated with age 3 psychological abuse (β = −0.07, *p* = 0.001). At age 5, substance use (β = 0.05, *p* = 0.008) and exposure to community violence (β = 0.11, *p* < 0.001) were positively associated with psychological abuse. At age 9, economic hardship (β = 0.04, *p* = 0.036), intimate partner violence (β = 0.07, *p* = 0.043), depression (β = 0.05, *p* = 0.010), and substance use (β = 0.05, *p* = 0.005) were positively associated with psychological abuse. In addition to these concurrent associations, several lagged effects of family and neighborhood contexts on psychological abuse were revealed. Economic hardship and intimate partner violence at age 3 were positively associated with psychological abuse at age 5 (economic hardship β = 0.12, *p* < 0.001; intimate partner violence: β = 0.09, *p* = 0.008) and age 9 (economic hardship β = 0.06, *p* = 0.005; intimate partner violence: β = 0.10, *p* = 0.001). Further, substance use at age 3 was positively associated with psychological abuse at age 5 (β = 0.04, *p* = 0.028) and substance use at age 5 was positively associated with psychological abuse at age 9 (β = 0.05, *p* = 0.006).

## 4. Discussion

The current study sought to understand the importance of family and neighborhood factors on child abuse risk at three stages of child development—early childhood, young school age, and middle childhood. Three family context variables had significant impacts on physical and psychological abuse, regardless of child developmental stage, economic hardship, maternal substance use, and IPV.

Early experiences of economic hardship were especially salient for the risk of physical and psychological abuse. Economic hardship reported when the focal child was age 3 was related to higher reported physical and psychological abuse at ages 3, 5, and 9, demonstrating both an immediate and lasting impact of early hardship on both types of maltreatment. The within-time finding coincides with prior research demonstrating a link between poverty and child maltreatment risk [6].

Parental substance use was also found to be critically important for child maltreatment risk. Maternal substance use at child age 3 was associated with increased risk for psychological abuse at ages 3 and 5, while substance use at child age 5 was associated with increased risk for both physical and psychological abuse at age 5 and psychological abuse at age 9, and substance use at child age 9 was associated with increased risk for both physical and psychological abuse at age 9. These findings fit with a large body of research finding an association between substance use and child maltreatment [16,26]. The longitudinal relationship between maternal substance use and abuse risk may be due to damages to the brain from long-term use of drug and alcohol [27,28] or due to decreased inhibitions during periods of use [15].

IPV was the final family-level factor that was found to have longitudinal impacts on child abuse risk. Within early childhood, it is related to increased risk for both physical and psychological abuse. IPV experienced at child age 3 is also associated with an increased risk of psychological abuse at ages 5 and 9, demonstrating the lasting impacts that violence between partners can have on parenting. These findings are consistent with work from Zolotor and colleagues [12], which found that rates of physical and psychological abuse were much higher in homes where IPV was present.

Maternal depression was found to have differential impacts based on the developmental stage of the child. Specifically, while we did not find significant associations between maternal depression and abuse at ages 3 or 5, we found a cross-sectional relationship between maternal depression and psychological abuse at child age 9. Our result is similar to another past study with a national sample of 2386 children that also identified the link between maternal depression and psychological abuse, but not physical abuse [14]. Despite mothers experiencing depression, children may be buffered against child maltreatment by mothers receiving social support from other family members [29]. On the other hand, having more children in the family increased the risk of child maltreatment [16]. Many children in FFCWS may have received nurturing from their grandparents in early childhood [30] that shielded against maternal depression perpetrating psychological abuse. Nonetheless, as time goes on, mothers may have lost social support, such as losing relationships with paternal grandparents due to relationship instability between biological mothers and fathers of children [30]. Furthermore, a potential increased number of siblings as the child grows up may inflict stress on mothers experiencing depression, which may have perpetuated psychological abuse against their children.

In terms of neighborhood context, we found evidence of impact on child abuse. At the study outset, we had hypothesized that neighborhood variables may have more of an impact when children were younger compared to older ages, specifically that the context would matter most when children were in early childhood (age 3), and then matter progressively less as children went on to ages 5 and 9. We had mixed evidence to support this hypothesis. Informal social control was in line with our hypothesis; specifically, we found that informal social control was protective against psychological abuse at child age 3, but did not find similarly protective effects at ages 5 or 9. We did not find social cohesion to be significantly related to physical or psychological abuse at any of the three time points. Although prior research has found a linkage with social cohesion and child maltreatment, it may be more directly related to child neglect than child abuse [31].

On the other hand, exposure to community violence was found to be especially salient at all three time points. Specifically, age 3 community violence was related to higher levels of both physical and psychological abuse at age 3 and more physical abuse at age 9. At age 5, only the within-time relationship was significant—age 5 exposure to community violence was related to higher levels of both physical and psychological abuse. The findings surrounding community violence provide support against our original hypothesis that community factors would matter less as children grow older. Community violence seems to be an especially important community context factor to consider in understanding how neighborhood conditions relate to parenting behaviors. It is possible that while parents may rely less on their neighbors for support as their children grow older, concerns about safety within the direct environment surrounding the home still come into play in significant ways in parenting choices and behaviors. Perhaps parents of children in violent communities use harsher forms of physical discipline to protect them from becoming victims of the violence within their neighborhood, and this phenomenon is not unique to specific developmental stages.

### 4.1. Limitations

This study has several limitations that must be considered. First, we relied upon secondary data that were collected from urban cities in the United States. The extent to which the findings extend to suburban and rural contexts is unknown. Second, all variables included were self-reported. Many of the constructs we sought to examine were sensitive in nature (e.g., child maltreatment, substance use), and therefore responses are subject to social desirability bias. Third, the data included an oversample of unmarried mothers by design. As such, the sample is very racially diverse and relatively lower income than the general population. Findings may not extend to other groups. Fourth, we focused on family and neighborhood factors as focal predictor of child abuse, yet other factors, such as biological and genetic factors, school and peer relationships, and cultural context, likely contribute to child maltreatment across different developmental stages of children. Future research may benefit from examining the extent to which these various factors across multiple levels of the social ecology influence abuse risk and child development. Finally, we focused only on child ages 3, 5, and 9 due to lack of reliable maltreatment data at child age 15 within the dataset.

### 4.2. Implications

This study offers several important implications for policy and practice. The lasting impact of early experiences of economic hardship suggests that anti-poverty policies and concrete supports to parents, especially those of young children, are crucial for child maltreatment prevention strategies. Further, given both the cross-sectional and longitudinal relationships between substance use and child abuse, child maltreatment prevention programs should screen for substance-use problems and assist families in reducing these conditions. The concurrent and lasting effects of maternal IPV on child abuse risk suggest that providing resources to IPV survivors to help them escape violent situations and holding abusers accountable are critical strategies to preventing child maltreatment from early to middle childhood. Finally, given that exposure to community violence was a salient risk factor for child abuse regardless of the developmental stage of the child, reducing exposure to crime within neighborhoods to make parents feel safer is likely to have a significant impact on reducing child abuse.

## 5. Conclusions

Overall, this study suggests that early intervention is key for preventing child maltreatment across the developmental stages of children. We found significant impacts of both family and neighborhood factors that lasted from early childhood into middle childhood. Future research studies should examine the specific mediators and moderators of these factors, to understand the pathways through which these factors relate to child maltreatment across developmental stages and the potential buffers of the risk factors and promoters of the risk factors.

## Figures and Tables

**Table 1:** Sample characteristics.

		%	M (SD)	Range
Child Characteristics				
	Sex (boys)	52.2%		
Race/Ethnicity				
	White; non-Hispanic	16.0%		
	Black; non-Hispanic	44.1%		
	Hispanic	22.9%		
	Multiracial	2.0%		
	Other	15.0%		
Maternal Characteristics				
	Age (in years)		25.28 (6.04)	15–43
	Educational level (high school or more)	72.1%		
	Married to the child’s father	32.1%		
Economic Hardship			1.72 (2.78)	0–9

**Table 2:** Time-varying predictors of physical abuse.

		Age 3 Physical Abuse			Age 5 Physical Abuse			Age 9 Physical Abuse		
		B	SE	*p*	B	SE	*p*	B	SE	*p*
Age 3										
	Economic hardship	**0.13**	**0.02**	**<0.001**	**0.08**	**0.02**	**<0.001**	**0.07**	**0.02**	**0.002**
	Intimate partner violence	**0.07**	**0.03**	**0.008**	0.06	0.03	0.063	−0.01	0.03	0.863
	Depression	0.03	0.02	0.051	0.03	0.02	0.194	0.01	0.02	0.875
	Substance use	0.03	0.02	0.119	0.01	0.02	0.700	0.01	0.02	0.650
	Social cohesion	−0.03	0.02	0.080	0.01	0.02	0.823	−0.01	0.02	0.675
	Social control	−0.02	0.02	0.231	−0.04	0.02	0.102	−0.01	0.02	0.506
	Community violence	**0.06**	**0.02**	**0.001**	0.01	0.02	0.560	**0.06**	**0.02**	**0.007**
Age 5										
	Economic hardship	─	─	─	0.03	0.02	0.156	−0.01	0.02	0.545
	Intimate partner violence	─	─	─	−0.02	0.03	0.601	0.01	0.04	0.745
	Depression	─	─	─	−0.03	0.02	0.148	0.03	0.02	0.090
	Substance use	─	─	─	**0.04**	**0.02**	**0.031**	0.03	0.02	0.165
	Social cohesion	─	─	─	−0.04	0.02	0.064	−0.02	0.02	0.394
	Social control	─	─	─	−0.03	0.02	0.205	−0.02	0.02	0.390
	Community violence	─	─	─	**0.06**	**0.02**	**0.001**	0.01	0.02	0.578
Age 9										
	Economic hardship	─	─	─	─	─	─	**0.05**	**0.02**	**0.006**
	Intimate partner violence	─	─	─	─	─	─	0.02	0.04	0.524
	Depression	─	─	─	─	─	─	0.02	0.02	0.375
	Substance use	─	─	─	─	─	─	**0.04**	**0.02**	**0.031**
	Social cohesion	─	─	─	─	─	─	−0.04	0.02	0.062
	Social control	─	─	─	─	─	─	0.03	0.02	0.135
	Community violence	─	─	─	─	─	─	0.02	0.02	0.277

Notes: Standardized parameter estimates are presented. Bolded numbers indicate statistically significant findings.

**Table 3:** Time-varying predictors of psychological abuse.

		Age 3 Psychological Abuse			Age 5 Psychological Abuse			Age 9 Psychological Abuse		
		B	SE	*p*	B	SE	*p*	B	SE	*p*
Age 3										
	Economic hardship	**0.12**	**0.02**	**<0.001**	**0.12**	**0.02**	**<0.001**	**0.06**	**0.02**	**0.005**
	Intimate partner violence	**0.11**	**0.03**	**<0.001**	**0.09**	**0.03**	**0.008**	**0.10**	**0.03**	**0.001**
	Depression	**0.05**	**0.02**	**0.002**	0.04	0.02	0.066	0.02	0.02	0.224
	Substance use	**0.04**	**0.02**	**0.009**	**0.04**	**0.02**	**0.028**	0.03	0.02	0.070
	Social cohesion	0.01	0.02	0.495	−0.01	0.02	0.508	−0.03	0.02	0.145
	Social control	**−0.07**	**0.02**	**0.001**	−0.03	0.02	0.137	−0.02	0.02	0.302
	Community violence	**0.07**	**0.02**	**<0.001**	0.01	0.02	0.619	0.01	0.02	0.679
Age 5										
	Economic hardship	─	─	─	0.02	0.02	0.427	−0.01	0.02	0.581
	Intimate partner violence	─	─	─	−0.03	0.03	0.342	−0.02	0.04	0.615
	Depression	─	─	─	0.03	0.02	0.148	0.03	0.02	0.088
	Substance use	─	─	─	**0.05**	**0.02**	**0.008**	**0.05**	**0.02**	**0.006**
	Social cohesion	─	─	─	0.02	0.02	0.321	0.02	0.02	0.308
	Social control	─	─	─	−0.03	0.02	0.111	−0.03	0.02	0.168
		─	─	─	**0.11**	**0.02**	**<0.001**	0.02	0.02	0.238
Age 9	Community violence									
	Economic hardship	─	─	─	─	─	─	**0.04**	**0.02**	**0.036**
	Intimate partner violence	─	─	─	─	─	─	**0.07**	**0.03**	**0.043**
	Depression	─	─	─	─	─	─	**0.05**	**0.02**	**0.010**
	Substance use	─	─	─	─	─	─	**0.05**	**0.02**	**0.005**
	Social cohesion	─	─	─	─	─	─	−0.01	0.02	0.518
	Social control	─	─	─	─	─	─	0.01	0.02	0.861
	Community violence	─	─	─	─	─	─	0.01	0.02	0.603

Notes: Standardized parameter estimates are presented. Bolded numbers indicate statistically significant findings.

## Data Availability

The data are publicly available through Fragile Families. https://fragilefamilies.princeton.edu, accessed on 15 December 2021.
